# Supplementary material for: Mediator kinase inhibition impedes transcriptional plasticity and prevents resistance to ERK/MAPK-targeted therapy in KRAS-mutant cancers
Source: NPJ Precis Oncol. 2024 May 31;8:124. doi: 10.1038/s41698-024-00615-9 (PMC11143207; doi:10.1038/s41698-024-00615-9)
Supplement: Supplementary file 1 — REPORTING SUMMARY [file 41698_2024_615_MOESM1_ESM.pdf]

nature portfolio

Corresponding author(s): Dr Kris Wood

Last updated by author(s): Mar 4, 2024

Reporting Summary

Nature Portfolio wishes to improve the reproducibility of the work that we publish. This form provides structure for consistency and transparency in reporting. For further information on Nature Portfolio policies, see our Editorial Policies and the Editorial Policy Checklist.

Statistics

For all statistical analyses, confirm that the following items are present in the figure legend, table legend, main text, or Methods section.

n/a	Confirmed	
☐	☒	The exact sample size (*n*) for each experimental group/condition, given as a discrete number and unit of measurement
☐	☒	A statement on whether measurements were taken from distinct samples or whether the same sample was measured repeatedly
☐	☒	The statistical test(s) used AND whether they are one- or two-sided<br>*Only common tests should be described solely by name; describe more complex techniques in the Methods section.*
☒	☐	A description of all covariates tested
☐	☒	A description of any assumptions or corrections, such as tests of normality and adjustment for multiple comparisons
☐	☒	A full description of the statistical parameters including central tendency (e.g. means) or other basic estimates (e.g. regression coefficient) AND variation (e.g. standard deviation) or associated estimates of uncertainty (e.g. confidence intervals)
☐	☒	For null hypothesis testing, the test statistic (e.g. *F*, *t*, *r*) with confidence intervals, effect sizes, degrees of freedom and *P* value noted<br>*Give P values as exact values whenever suitable.*
☒	☐	For Bayesian analysis, information on the choice of priors and Markov chain Monte Carlo settings
☒	☐	For hierarchical and complex designs, identification of the appropriate level for tests and full reporting of outcomes
☐	☒	Estimates of effect sizes (e.g. Cohen's *d*, Pearson's *r*), indicating how they were calculated

Our web collection on statistics for biologists contains articles on many of the points above.

Software and code

Policy information about availability of computer code

Data collection	-Cell Counting: Z2 Coulter Particle Count and Size Analyzer<br>-Plate Reader for luminescence assays: TECAN infinite M1000Pro<br>-DNA/RNA concentration measurement: Thermo Fisher NanoDrop Lite<br>-Blot image scanning: Bio-Rad ChemiDoc MP System<br>-RNA and DNA sequencing: Illumina HiSeq and NextSeq<br>-RPPA: 2470 Aushon arrayer
Data analysis	-Statistical analysis and plotting were done in GraphPad Prism 8.0.2, 9.0.1 or Microsoft Excel 2016-2021<br>-Figures were prepared in Microsoft PowerPoint<br>-RNA seq was analyzed using DESeq2 Bioconductor package with R

For manuscripts utilizing custom algorithms or software that are central to the research but not yet described in published literature, software must be made available to editors and reviewers. We strongly encourage code deposition in a community repository (e.g. GitHub). See the Nature Portfolio guidelines for submitting code & software for further information.

Data

Policy information about availability of data

All manuscripts must include a data availability statement. This statement should provide the following information, where applicable:

- Accession codes, unique identifiers, or web links for publicly available datasets
- A description of any restrictions on data availability
- For clinical datasets or third party data, please ensure that the statement adheres to our policy

All data associated with this study are available in the main text or the supplementary materials. Source data are provided with this paper. RNA-seq and ChIP-seq raw data have been made available at Gene Expression Omnibus (GEO). For ChIP-seq data, the GEO accession number is GSE234378. For RNA-seq data, the GEO accession number is GSE237177.

Research involving human participants, their data, or biological material

Policy information about studies with human participants or human data. See also policy information about sex, gender (identity/presentation), and sexual orientation and race, ethnicity and racism.

Reporting on sex and gender	N/A
Reporting on race, ethnicity, or other socially relevant groupings	N/A
Population characteristics	N/A
Recruitment	N/A
Ethics oversight	N/A

Note that full information on the approval of the study protocol must also be provided in the manuscript.

Field-specific reporting

Please select the one below that is the best fit for your research. If you are not sure, read the appropriate sections before making your selection.

☒ Life sciences ☐ Behavioural & social sciences ☐ Ecological, evolutionary & environmental sciences

For a reference copy of the document with all sections, see nature.com/documents/nr-reporting-summary-flat.pdf

Life sciences study design

All studies must disclose on these points even when the disclosure is negative.

Sample size	As described, in vitro sample sizes ranged from n=3 to n=10 replicates per condition. Sample sizes were sufficient to identify significant changes as indicated in each figure.
Data exclusions	No data were excluded from analysis.
Replication	Experimental findings were performed at least 2 to 3 independent times as indicated in the figure legends and methods. The experimental findings were reliably reproduced and all attempts were included in the presentation unless technical error prevented the completion of the experiment.
Randomization	Mice were randomized prior to initiation of study treatment. No randomization was required for the in vitro cell line experimentation.
Blinding	Blinding the in vivo experiments was not feasible given the labeling requirements of the facilities and limitation of available personnel. To establish humane endpoint for mouse study, blinded observers visually inspected mice for obvious signs of distress, such as loss of appetite, hunched posture. No blinding of in vitro experiments was performed as each cell line requires different culture conditions.

Reporting for specific materials, systems and methods

We require information from authors about some types of materials, experimental systems and methods used in many studies. Here, indicate whether each material, system or method listed is relevant to your study. If you are not sure if a list item applies to your research, read the appropriate section before selecting a response.

Materials & experimental systems

n/a	Involved in the study	
☐	☒	Antibodies
☐	☒	Eukaryotic cell lines
☒	☐	Palaeontology and archaeology
☐	☒	Animals and other organisms
☒	☐	Clinical data
☒	☐	Dual use research of concern
☒	☐	Plants

Methods

n/a	Involved in the study	
☐	☒	ChIP-seq
☒	☐	Flow cytometry
☒	☐	MRI-based neuroimaging

Antibodies

Antibodies used	KRAS (CST #14429), p-MEK (CST #9121), T-MEK (CST #4694), p-ERK (CST #9101), T-ERK (CST#4695), vinculin (CST#4650), CDK8 (CST #4106), CDK19 (Sigma HPA007053), H3K27ac (Active Motif, Catalog number: 39133)
Validation	Antibody validation of the manufacturers were available for all antibodies including positive and negative staining controls. CDK8 and CDK19 antibodies were additionally validated through knockout experiments.

Eukaryotic cell lines

Policy information about cell lines and Sex and Gender in Research

Cell line source(s)	All human cell lines were purchased from American Type Culture Collection (ATCC) or Duke University Cell Culture Facility (CCF). The commonly-used cancer cell lines used in this study were MIA PaCa-2, SW1573, SW620, CFPAC1, AsPC-1.
Authentication	All cell lines were authenticated prior to use using STR profiling.
Mycoplasma contamination	All cell lines were confirmed as mycoplasma-free upon receipt.
Commonly misidentified lines (See ICLAC register)	No cell lines were misclassified.

Animals and other research organisms

Policy information about studies involving animals; ARRIVE guidelines recommended for reporting animal research, and Sex and Gender in Research

Laboratory animals	FVB/N mice, 8 weeks old.
Wild animals	No wild animals were used in this study.
Reporting on sex	Only female mice were used for this study.
Field-collected samples	No samples collected from the field were used in this study.
Ethics oversight	The University of North Carolina Institutional Animal Care & Use Committee (IACUC) reviewed and approved the protocol described in this study.

Note that full information on the approval of the study protocol must also be provided in the manuscript.

Plants

Seed stocks	N/A
Novel plant genotypes	N/A
Authentication	N/A

ChIP-seq

Data deposition

☒ Confirm that both raw and final processed data have been deposited in a public database such as GEO.

☒ Confirm that you have deposited or provided access to graph files (e.g. BED files) for the called peaks.

Data access links
May remain private before publication.

https://urldefense.com/v3/__https://www.ncbi.nlm.nih.gov/geo/query/acc.cgi?acc=GSE234378__;!!OToaGQ!thJkWG87o5lSu_IgncpS5d6_9rh1GliTr8GWnLOzbCzVGZLb1U2PQZwkA6eCbFBPxbubkjP41VliglQUoL7p6-9BvA$

GEO accession: GSE234378
Reviewer token: kfwxecqopvqjvan

Files in database submission

GSM7466420_hg38.H3K27ac.1W.rep1.masked.dedup.sorted.rpkm.ctrl_subtracted.bw
GSM7466420_hg38.H3K27ac.1W.rep1.masked.dedup.sorted_peaks.narrowPeak.gz
GSM7466422_hg38.H3K27ac.1W.rep2.masked.dedup.sorted.rpkm.ctrl_subtracted.bw
GSM7466422_hg38.H3K27ac.1W.rep2.masked.dedup.sorted_peaks.narrowPeak.gz
GSM7466424_hg38.H3K27ac.Control.rep1.masked.dedup.sorted.rpkm.ctrl_subtracted.bw
GSM7466424_hg38.H3K27ac.Control.rep1.masked.dedup.sorted_peaks.narrowPeak.gz
GSM7466426_hg38.H3K27ac.Control.rep2.masked.dedup.sorted.rpkm.ctrl_subtracted.bw
GSM7466426_hg38.H3K27ac.Control.rep2.masked.dedup.sorted_peaks.narrowPeak.gz
GSM7466428_hg38.H3K27ac.Resistant.rep1.masked.dedup.sorted.rpkm.ctrl_subtracted.bw
GSM7466428_hg38.H3K27ac.Resistant.rep1.masked.dedup.sorted_peaks.narrowPeak.gz
GSM7466430_hg38.H3K27ac.Resistant.rep2.masked.dedup.sorted.rpkm.ctrl_subtracted.bw
GSM7466430_hg38.H3K27ac.Resistant.rep2.masked.dedup.sorted_peaks.narrowPeak.gz

Genome browser session
(e.g. UCSC)

https://urldefense.com/v3/__https://www.ncbi.nlm.nih.gov/geo/query/acc.cgi?acc=GSE234378__;!!OToaGQ!thJkWG87o5lSu_IgncpS5d6_9rh1GliTr8GWnLOzbCzVGZLb1U2PQZwkA6eCbFBPxbubkjP41VliglQUoL7p6-9BvA$

Methodology

Replicates

Two replicates per condition.

Sequencing depth

18M reads per sample, x3 samples each in triplicate. Sequenced using 75bp single-end reads.

Antibodies

H3K27ac
Supplier: Active Motif
Catalog number: 39133
Clone name: Polyclonal

Peak calling parameters

Adapter sequences were removed from the raw reads using Trimmomatic v0.32 (PMID: 24695404). Reads were aligned using Bowtie v1.0.0 (PMID:19261174), reporting the best alignment with up to 2 mismatches (parameters --best --strata -v 2 -X 2000). Duplicates were marked using Picard MarkDuplicates v1.130 (http://broadinstitute.github.io/picard/), while low mappability or blacklisted regions identified by the ENCODE project were filtered out from the final BAM files. Signal files were generated with deeptools bamCoverage (v3.0.1 PMID:24799436) ignoring duplicates, extending reads 200bp and applying RPKM normalization. Using the sequenced input controls, binding regions were identified using the callpeak function in MACS2 v2.1.1.20160309 (PMID:18798982) after estimating the fragment size using the strand cross-correlation method (parameters: --broad --extsize <ESTIMATED_FRAG_SIZE> --nomodel).

Data quality

Peaks were first identified using MACS2 with a configuration suitable to detect narrow peaks as those typically observed in H3K27ac data. A union peakset of all possible acetylation events identified across conditions was then defined. Using this common set, reads in peaks were computed using featureCounts with default parameters. Lastly, to detect differential binding events a negative binomial model using DESeq2 was applied to the counts, followed by a Wald test to compute adjusted p-values. Gained peaks represent both regions gaining or increasing acetylation when compared to control samples. Conversely, sites with depleted or decreased signal are referred to as lost peaks. In total, 1932 differential peaks were identified with an adjusted FDR <5%. Of these, 109 peaks demonstrated >5-fold enrichment or loss (|log2fold change| > 2.321928).

Software

Trimmomatic, Bowtie, Picard MarkDuplicates, bamCoverage, MACS2, featureCounts, DESeq2
